# Study of the Nanofibers Fabrication Conditions from the Mixture of Poly(vinyl alcohol) and Chitosan by Electrospinning Method

**DOI:** 10.3390/polym14040811

**Published:** 2022-02-19

**Authors:** Thi Hong Nhung Vu, Svetlana N. Morozkina, Mayya V. Uspenskaya

**Affiliations:** Chemical Engineering Centre, ITMO University, Kronverkskiy Prospekt, 49A, 197101 St. Petersburg, Russia; i_norik@mail.ru (S.N.M.); mv_uspenskaya@mail.ru (M.V.U.)

**Keywords:** biomaterials, electrospinning, fiber technology, poly (vinyl alcohol), chitosan

## Abstract

Nanofiber fabrication is attracting great attention from scientists and technologists due to its applications in many fields of life. In order to design a nanosized polymer-based drug delivery system, we studied the conditions for the fabrication of electrospun nanofibers from poly (vinyl alcohol) (PVA) and chitosan (CS), which are well-known as biocompatible, biodegradable and non-toxic polymers that are widely used in the medical field. Aiming to develop nanofibers that can directly target diseased cells for treatment, such as cancerous cells, the ideal choice would be a system that contains the highest CS content as well as high quality fibers. In the present manuscript, it is expected to become the basis for improving the low bioavailability of medicinal drugs limited by poor solubility and low permeability. PVA–CS nanofibers were obtained by electrospinning at a PVA:CS ratio of 5:5 in a 60% (*w*/*w*) acetic acid solution under the following parameters: voltage 30 kV, feed rate 0.2 mL/h, needle-collector distance 14 cm. The obtained fibers were relatively uniform, with a diameter range of 77–292 nm and average diameter of 153 nm. The nanofiber system holds promise as a potential material for the integration of therapeutic drugs.

## 1. Introduction

In recent years, electrospinning technology has gained more and more attention along with the development of nanotechnologies, which have been used by scientists and technicians in a wide variety of scientific and technological fields. It is important to mention that healthcare has been one of the pioneers in the development of nanotechnologies, with applications in such areas as fluorescent biological labeling, drug and gene delivery, bio-detection of pathogens, detection of proteins, probing of DNA structure, tissue engineering, tumor detection, separation and purification of biological molecules and cells, MRI contrast enhancement and pharmacokinetic studies [1]. Nano-sized drug capsules also have gained increasing attention due to the fact that they are believed to have outstanding advantages over conventional sized capsules. Given the same drug mass, nano-sized capsules have a significantly higher total surface area as well as a higher drug decomposition and absorption rates. Another nanomaterial that exhibits outstanding advantages is electrospun fibers, which are used as a drug carriers. Due to their size, nano-sized capsules allow otherwise difficult-to-absorb drugs to be absorbed and slowly distributed into the body, thus enhancing their absorption and providing better therapeutic effect. In addition, biodegradable materials used as drug carriers can be broken down into small molecules that are absorbed or excreted from the body; during this process, the drug can be easily released and assimilated, improving its therapeutic function [2]. The fabrication of nanofibers by the electrospinning technique follows the fundamental principle of fabricating nanofibers from molten polymers or polymer solutions under the effect of high-voltage current. In that process, the polymer solution is streamed under the action of a high-voltage electric field; while being ejected from the needle tip, the solution adopts a Taylor cone shape, then changes into a liquid jet that solidifies into nanofibers over the collector’s surface [3,4].

**Figure 1:**
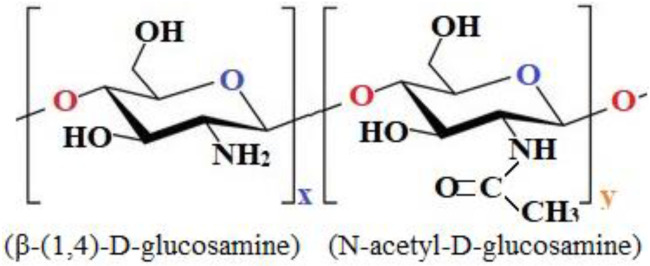
Molecular structure of chitosan.

Chitosan is a linear polysaccharide composed of randomly distributed deacetylated units (β-(1,4)-D-glucosamine) and acetylated units (N-acetyl-D-glucosamine) (Figure 1). Chitosan is obtained by deacetylation from chitin, which is the second most common polysaccharide in the world, only after cellulose. Usually, chitin with more than 60% degree of deacetylation [5,6] is considered as chitosan; the degree of deacetylation of chitosan is in the range of 60–98% [7]. Known as an attractive material for medical use, chitosan possesses excellent biological properties such as biodegradability, lack of toxicity, antifungal effects, and the ability to accelerate wound healing and stimulate the immune system [8,9]. Due to its ability to function in a variety of forms, chitosan has attracted much attention in the fields of orthopedics and periodontitis therapy [10,11], tissue engineering [10,11,12], wound healing [10,12], and drug transport [10]. In biomedical applications, it has been used for artificial skin, surgical sutures, artificial blood vessels, controlled drug release, contact lenses, eye moisturizers, bandages, sponges, cholesterol control, anti-inflammatory treatment, tumor inhibition, antiviral drugs, inhibition of plaque, bone healing, accelerated wound healing; hemostatic, antibacterial, and antifungal applications; and weight loss effects [10]. The reason for our preference for chitosan as a nanofiber-forming material was the distribution of amino groups along the molecule, as well as its polysaccharide structure. The amino groups can be protonated, providing the chitosan with different properties such as passing through characteristic neutralization reactions of alkaline compounds [8] and ensuring solubility in dilute acidic aqueous solutions (pH < 6) [5,13]. It is known that pathological microenvironments are mostly acidic: for example, inflammation-associated acidic pH of 7.2–6.5 for the extracellular pH in tumors; pH 5.4 in inflamed tissue; pH 4.7 in fracture-related hematomas; and pH 5.7 in cardiac ischemia [14,15]. Thus, the base structure of chitosan will help the formed nanofibers to be highly targeted to pathological cells and tissues. As a polycationic polymer with a high density of positive charges, it can adhere to both hard and soft tissues such as epithelial and mucous tissues through hydration, hydrogen bonding and ionic interactions, and has been extensively investigated as a drug carrier for targeted drug delivery purposes [16]. Cancerous cells are characterized by an abnormal glucose metabolism pathway that leads to the need for high glucose to rapidly generate energy for their survival [17,18,19]. Chitosan has been shown to be suitable as a material to target cancerous cells due to its polysaccharide structure. In addition, as a polysaccharide that contains degradable glycosidic bonds, chitosan is biodegradable by several protease enzymes, and mainly by lysozyme [5,13]. Chitosan is a biopolymer that behaves like a hydrogel due to its three-dimensional structure, which can absorb and retain large amounts of water, allowing it to swell without the need for complete dissolution [7]. In solution, chitosan macromolecules tend to entangle and form physical networks due to the abundant intermolecular hydrogen bonding, even at low concentrations. This causes some difficulties in the fabrication of nanofibers with only chitosan, requiring that it be combined with another polymer that has more favorable properties for nanofiber fabrication; PVA was chosen for our investigation.

**Figure 2:**
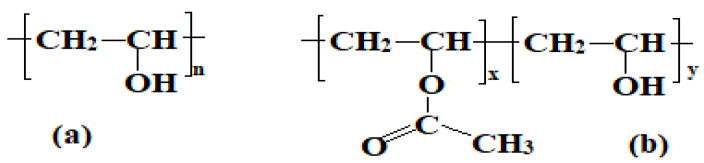
Molecular structure of poly (vinyl alcohol). (**a**) fully hydrolyzed PVA and (**b**) partially hydrolyzed PVA.

Poly (vinyl alcohol) (PVA) (Figure 2) has attracted considerable research interest and is recognized as one of the most extensively produced synthetic polymers worldwide [20]. PVA is a widely used thermoplastic polymer that is safe for living tissues, harmless and non-toxic. Orally administered poly (vinyl alcohol) has an LD_50_ ≈ 15–20 g/kg [21,22]. PVA is a semicrystalline synthetic polymer, which is soluble in water, slightly soluble in ethanol and insoluble in other organic solvents [22,23,24,25]. It is also a biodegradable polymer, and its degradability is enhanced by hydrolysis due to the presence of hydroxyl groups. Under the action of the microbial community or some enzymes such as *ß-diketone hydrolase* and *secondary alcohol oxidase*, the PVA molecular chain can be partially cleaved at C–C bonds to form ketone and carboxylic compounds [26,27,28]. The final breakdown product during PVA degradation is acetic acid, which is transferred into the central metabolic pathway. Acetic acid is readily metabolized by most human and animal tissues. It is also involved in the formation of phospholipids, neutral lipids, sterols, and saturated and unsaturated fatty acids in many human and animal tissues [29,30].

PVA is commercially produced through the hydrolysis of poly (vinyl acetate) in a two-step process consisting of free radical polymerization of vinyl acetate followed by its hydrolysis [20,31]. Therefore, the structural properties of PVA are mainly dependent on the molecular weight of the polymer and the degree of hydrolysis, i.e., the percentage of vinyl alcohol in the polymer [31,32]. As the degree of vinyl acetate hydrolysis into vinyl alcohol increases, the polymer structure becomes more crystalline, which results in a highly durable PVA structure, which becomes chemically inert [31]. The degree of crystallinity plays a major role in controlling the diffusion of drugs from PVA hydrogels [33], which can be designed either as matrix or reservoir for drug delivery platforms [34]. In general, due to its biocompatibility, drug compatibility, water solubility, film formation, mechanical properties and good swelling, PVA has been studied as a material for ocular inserts, ocular films, nanoparticles, microspheres, floating microspheres, mucoadhesives, transdermal patches, and intramuscular drug delivery systems, as well as targeted drug delivery for colon, rectal, buccal, transdermal, pH- and temperature-sensitive drug delivery systems and swelling-controlled drug delivery systems [20,31,35]. Due to its susceptibility to hydrogen bonding and excessive crystallization, PVA is very sensitive to moisture; PVA hydrogels generally have low mechanical properties and offer very low swelling capacity, making them desirable only for specific biomedical and pharmaceutical applications [31,36,37]. In order to improve its mechanical properties and stability, polyvinyl alcohol is often mixed with other biopolymers. It has been reported that the inclusion of chitosan into a polyvinyl alcohol matrix can improve its biocompatibility and mechanical properties [24,38,39].

To date, there have been many reports referring to the fabrication of electrospun PVA–chitosan nanofibers, from which the smallest fibers are reported to have an average diameter of 20 to 100 nm [39]. However, such fibers were interspersed with enlarged spindle-like sections of about 500 ± 100 nm in width. The addition of PVA facilitated the formation of chitosan nanofibers, but only when the chitosan content was equal or less than 25% [39]. In another report on a morphological study of PVA–CS, nanofibers were prepared by electrospinning and collected on reticulated vitreous carbon; nanofibers with diameters from 132 to 212 nm were obtained from a mixture of 8% PVA and 2% chitosan in 2% acetic acid solution [40]. PVA–CS nanofibers produced by the electrospinning technique were also obtained from an acetic acid solution with up to 70% concentration and PVA: CS ratio of 70:30 [41]. There are obviously major differences in PVA–CS nanofiber fabrication methods and results, so more detailed and specific studies are needed. In this study, we conducted our experiments step-by-step to estimate the correlation between the influence of the solution conditions and the electrospinning parameters on the formation of PVA–CS nanofibers.

## 2. Materials and Methods

### 2.1. Materials

CS (M_W_ 200 kDa) and PVA (M_W_ 55 kDa) were used as received from the Russian Federation limited liability company Bioprogres.

Acetic acid (99.5%) and distilled water were used as components of the binary solvent system.

### 2.2. Electrospinning Technique

**Figure 3:**
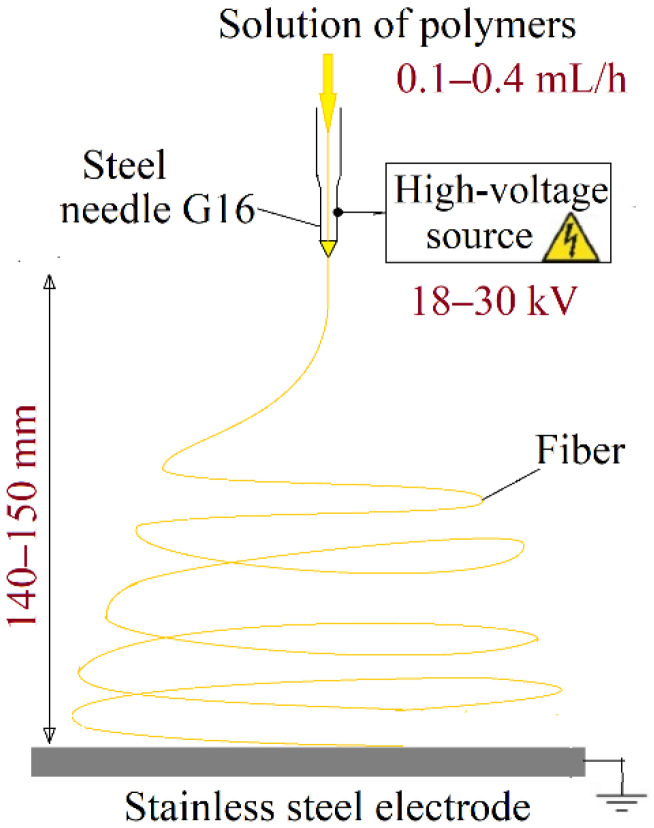
Schematic representation of the electrospinning process and operating parameters.

PVA–CS solutions were electrospun in a NANON-01A system (MECC CO., LTD., Fukuoka, Japan). The electrospinning process (Figure 3) was performed at the temperature of 28.0 ± 1.5 °C and the relative humidity of 20 ± 3%. The technical parameters were changed in order to find the optimal conditions for the production of fibers: voltage range from 18 to 30 kV; feed rate range from 0.1 to 0.4 mL/h; needle-collector distance switched between 140 mm and 150 mm; traverse speed 10 mm/s, 16G steel needles; plate stainless steel collector 150 mm × 200 mm (L × W).

The study of nanofiber fabrication included studying the influence of different PVA:CS ratios and an investigation of the solvent’s influence on the formation of nanofibers at different needle-collector distances, voltages, and feed rate speeds.

### 2.3. Rheological Properties of Polymer Solutions

An MCR 502 rheometer with a cylinder was utilized to obtain the kinematic viscosity of the solutions. Measurements were performed at a temperature of 25 °C and in a shear rate range from 0.1 to 500 s^−1^.

The conductivity of the polymer solution was measured on a WTW inoLab Cond 7110 conductivity meter with a WTW TetraCon 325 sensor. Measurement error should not exceed 0.5%.

### 2.4. Morphology and Diameters of Nanofibers

For the preliminary characterization of the morphology and diameters of the electrospun PVA–CS fibers, the optical microscope Olympus STM6 (OLYMPUS Corporation, Tokyo, Japan) was used. Differentially interferential contrasting technique (DIC) was applied to emphasize the colorfulness and contrast of the obtained fibers. ImageJ (National Institutes of Health, Bethesda, MD, USA) was used for the analysis and measurement of the fiber diameters from the obtained microphotograph program.

### 2.5. Infrared Spectroscopy

A Bruker alpha Fourier transform Infrared (FTIR) spectrometer (Bruker, Germany) was used to obtain the infrared absorption spectra of the samples.

### 2.6. Statistical Analysis

The diameter distribution of the obtained nanofibers was estimated by OriginPro 2019b (OriginLab Corporation, Northampton, MA, USA). To measure the diameter distribution, several images were used.

## 3. Results and Discussion

### 3.1. Investigation of the Influence of Acetic Acid Concentration on PVA–CS Nanofiber Fabrication

#### 3.1.1. Selection of PVA:CS Ratio to Investigate Nanofiber Fabrication Conditions

During the fabrication of PVA nanofibers from acetic acid solution, we found that the optimal parameters were 12% (*w*/*w*) PVA and 30% (*w*/*w*) CH_3_COOH with the rest as deionized water. In order to develop a nanofiber system that could integrate medicinal compounds, we added CS to PVA nanofibers. The first step for obtaining the PVA–CS fibers was to keep constant the CS concentration at 2% (*w*/*w*) and change the PVA concentration to find the optimal concentration for the electrospun fibers.

Polymer solutions were prepared by mixing 2% (*w*/*w*) CS with different concentrations of PVA in a 30% (*w*/*w*) acetic acid solution. All solutions were stirred at 90 °C until homogeneous. The electrospinning parameters used during the process were: feed rate range 0.1–0.4 mL/ h; voltage range 18–30 kV; and needle-collector distance of 140 and 150 mm. The results of nanofiber fabrication from the polymer solutions with different PVA:CS ratios are presented in Table 1.

A PVA: CS ratio of 8:2 was chosen for the investigation of the solvents and electrospinning conditions used to fabricate the nanofiber system.

#### 3.1.2. Influence of Acetic Acid Concentration on PVA–CS Nanofiber Fabrication

We studied the effect of CH_3_COOH concentration on fiber formation from the PVA:CS (8:2) polymer system, see Table 2

When the acid concentration was increased to 50% (*w*/*w*) and 60% (*w*/*w*), the solution easily formed nanofibers. However, when the concentration of acetic acid was increased to 70% (*w*/*w*), the solution became too viscous, which inhibited its use for electrospinning.

### 3.2. Determination of Electrospinning Parameters for PVA–CS Nanofiber Fabrication

The use of acetic acid at a concentration of 50% or 60% allows the formation of fibers with a PVA:CS ratio of 8:2. Therefore, the study on the formation of nanofibers from different PVA:CS ratios was carried out at both acetic acid concentrations.

Solutions with different PVA:CS ratios were dissolved in 50% (*w*/*w*) and 60% (*w*/*w*) CH_3_COOH. The solutions were prepared by stirring at 90 °C until a homogeneous solution was obtained. The electrospinning parameters were fixed as follows: needle-collector distance 140 mm, feed rate range 0.1–0.4 mL/h and voltage range 18–30 kV. The results of nanofiber fabrication from the polymer solutions at different PVA:CS ratios are presented in Table 3.

Solutions with the concentration of acetic acid at 60% (*w*/*w*) proved to be the most favorable for the fabrication of nanofibers. From the obtained results, it was possible to observe that the solution containing a higher concentration of PVA needed a lower feed rate and voltage, which eased the fabrication of fibers. However, lower feed rates and higher voltages resulted in polymer solutions that dried faster, causing clogging at the needle tip, which easily occurred especially with solutions with high chitosan concentrations above 4% (*w*/*w*) and 5% (*w*/*w*). Additionally, from visual observation, it could be seen that the solutions with a higher concentration of PVA resulted in easier fiber formation. The solution with a concentration of chitosan greater than 5% (*w*/*w*) was too viscous and could not be used for electrospinning. In order to choose the proper ratio of polymers for fabrication of nanofibers, the morphology of the fibers must be analyzed using microscopy techniques.

For further analysis, we selected all the samples obtained under the following parameters: needle-collector distance of 140 mm, voltage of 30 kV and feed rate of 0.2 mL/h.

### 3.3. Morphology and Size Distribution of PVA–CS Nanofibers

**Figure 4:**
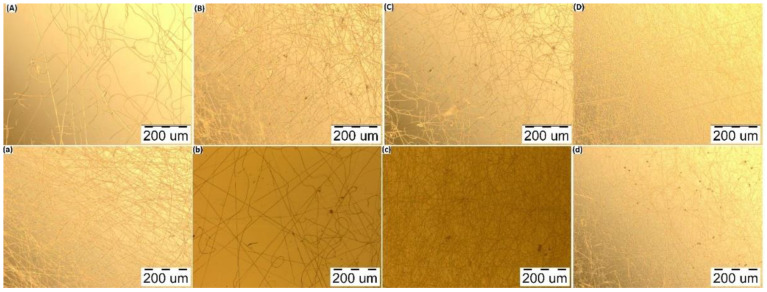
Micrographs of the electrospun fibers from the polymer solutions at the distance of 140 mm, flow rate 0.2 mL/h, voltage 30 kV (at 100× magnification). (**A**) PVA:CS = 8:2; (**B**) PVA:CS = 7:3; (**C**) PVA:CS = 6:4; (**D**) PVA:CS = 5:5 (CH_3_COOH 50%); (**a**) PVA:CS = 8:2; (**b**) PVA:CS = 7:3; (**c**) PVA:CS = 6:4; (**d**) PVA:CS = 5:5 (CH_3_COOH 60%).

**Figure 5:**
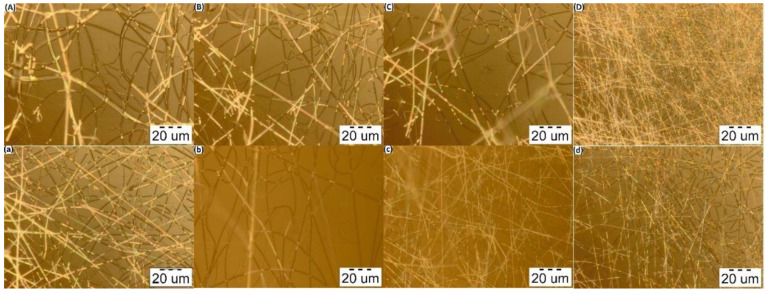
Micrographs of the electrospun fibers from the polymer solutions at the distance of 140 mm, flow rate 0.2 mL/h, voltage 30 kV (at 1000× magnification). (**A**) PVA:CS = 8:2; (**B**) PVA:CS = 7:3; (**C**) PVA:CS = 6:4; (**D**) PVA:CS = 5:5 (CH_3_COOH 50%); (**a**) PVA:CS = 8:2; (**b**) PVA:CS = 7:3; (**c**) PVA:CS = 6:4; (**d**) PVA:CS = 5:5 (CH_3_COOH 60%).

**Figure 6:**
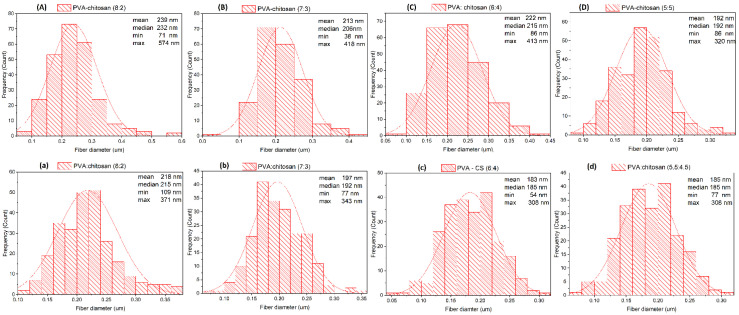
Diameter distribution diagram of PVA–CS fibers. (**A**) PVA:CS = 8:2; (**B**) PVA:CS = 7:3; (**C**) PVA:CS = 6:4; (**D**) PVA:CS = 5:5 (CH_3_COOH 50%); (**a**) PVA:CS = 8:2; (**b**) PVA:CS = 7:3; (**c**) PVA:CS = 6:4; (**d**) PVA:CS = 5:5 (CH_3_COOH 60%).

In order to select the proper polymer ratio, the influence that different polymer concentrations have on the formation and morphology of the fibers must be taken into consideration. PVA–CS solutions with ratios of 8:2; 7:3; 6:4 and 5:5 (%, *w*/*w*) and acetic acid concentrations at 50% (*w*/*w*) and 60% (*w*/*w*) were electrospun under the same parameters: voltage 30 kV, needle-collector distance 140 mm, and feed rate 0.2 mL/h. The morphological characteristics and diameter distribution of the obtained PVA–CS fibers are presented in Figure 4, Figure 5 and Figure 6, and Table 4.

From the nanofiber morphology investigation, it could be seen that by increasing the CS concentration to the point where PVA and CS concentrations were equal, the diameter of the fibers decreased; moreover, it was possible to observe an improvement in the fibers’ morphology. Therefore, for the fabrication of high-quality fibers, it is necessary to find the optimal polymer ratios as well as the optimal electrospinning parameters.

In summary, increasing the concentration of acetic acid not only improved the fabrication of the fibers, but also improved the morphology and decreased the diameters of the PVA–CS nanofibers. The higher the concentration of PVA used, the more easily that fibers were obtained, but also the more defects that appeared; on the other hand, higher CS concentrations required more precise electrospinning parameters but resulted in fibers with better morphology and increased nanoscale. When the CS concentration was higher than the PVA concentration (higher than 5% (*w*/*w*)), the solution became too viscous and could not form nanofibers. However, the ratio of 5% (*w*/*w*) CS together with 5% (*w*/*w*) PVA was higher if compared to previously published reports on the PVA:CS ratio for nanofiber fabrication, that is, in the range from 8:2 to 7:3 [39,40,41]. With the expectation of including as much CS in the PVA–CS nanofiber as possible, in order to increase the ability to integrate drugs and increase the targeting ability to treat diseased cells in the body, the 5:5 ratio is ideal.

Thus, it was determined that the optimal parameters for the production of high-quality nanofibers are: for the polymer solution, the optimal concentrations of polymers are 5% (*w*/*w*) PVA, 5% (*w*/*w*) CS, and 60% (*w*/*w*) acetic acid. For the electrospinning, the optimal parameters are: needle-collector distance—140 mm; feed rate—0.2 mL/h, and voltage—30 kV.

### 3.4. Investigation of Rheological Properties of PVA–CS Solutions 

The nanofiber fabrication ability as well as the nanofiber morphology are closely related to the rheological properties of the polymer solutions used. To understand this correlation, we studied the change in the conductivity and viscosity values of the polymer solutions at different PVA:CS ratios.

#### 3.4.1. Electrical Conductivity

**Figure 7:**
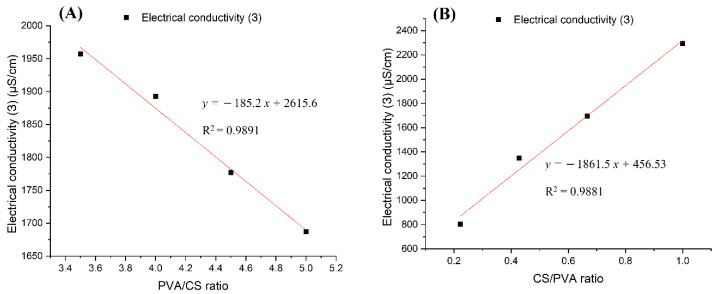
Diagrams of the electrical conductivity dependence on the ratio of polymers in the solutions: (**A**) PVA x%: CS 2% in 30% (*w*/*w*) acetic acid; (**B**) PVA x%: CS y% in acetic acid 60%.

The conductivity of the PVA–CS solutions was considered in two cases: first, the CS concentration was kept at 2% (*w*/*w*) while the PVA concentration was changed in 30% (*w*/*w*) acetic acid; in the second case, the concentrations of both PVA and CS were changed in 60% (*w*/*w*) acetic acid. The results are presented in Table 5 and Table 6 and Figure 7.

Considering the conductivity values of PVA:CS solutions with the ratio 9:2 in the above two cases, it was found that the increase in acetic acid concentration from 30% (*w*/*w*) to 60% (*w*/*w*) caused the conductivity to decrease from 1777 μS/cm to 802 μS/cm. The cause of the decrease in conductivity with the increased acetic acid concentration was due to the increase in the density and strength of the hydrogen bonds between the acetic acid and polymer molecules. It is these connections that increase the degree of entanglement of the polymers in solution and hinder the movement of charge carriers and free ions present in the solution.

By comparing the results presented in Figure 7A,B it is easy to conclude that while increasing the concentration of PVA decreased the conductivity of the solution, a higher concentration of CS led to an increase in solution conductivity. It can be concluded that the CS molecules have an extremely large charge carrier release, which overcomes the entanglement of the polymer system and the abundant hydrogen bonding in the solution.

#### 3.4.2. Viscosity

**Figure 8:**
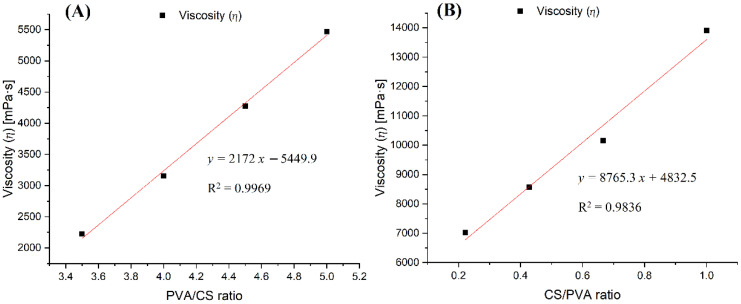
Diagram of the dependence of viscosity on the ratio of polymers in the solutions: (**A**) PVA x%: CS 2% in 30% (*w*/*w*) acetic acid; (**B**) PVA x%: CS y % in acetic acid 60% (*w*/*w*).

The viscosity of the PVA–CS solutions was studied. PVA–CS solutions were considered in two cases: first, the CS concentration was kept at 2% (*w*/*w*) while the PVA concentration was changed in 30% (*w*/*w*) acetic acid; in the second case, the concentrations of both PVA and CS were changed in 60% (*w*/*w*) acetic acid. Measurements were made at 25 °C and in a range of shear rates from 0.1 to 500 s^−1^; the results were obtained at a shear rate of 75.3 s^−1^ and are presented in Table 7 and Table 8 and Figure 8.

The viscosity values of the PVA:CS solutions with the ratio of 9:2 in the above two cases changed from 4275.6 mPa.s to 7016.4 mPa.s along with the increase in the concentration of acetic acid from 30% (*w*/*w*) to 60% (*w*/*w*). Similarly to the effect of acid concentration on electrical conductivity, the increase in viscosity in this case can also be explained by the increasing number of hydrogen bonds between the acetic acid and the polymers in the solution, thereby increasing the viscosity of solutions with higher concentrations of acetic acid.

An increase in the concentration of any polymer had the effect of increasing the solution’s viscosity. As illustrated in Figure 8A, when the concentration of CS was fixed at 2% (*w*/*w*) and the concentration of PVA was changed, it was observed that the viscosity of the solution was proportional to the increase in PVA concentration. When the concentration of CS was increased and the concentration of PVA decreased, the viscosity of the solution still increased very quickly, by up to about four-fold compared to case (Figure 8A,B). In addition, considering the difference in molecular weight between the two polymers, the increase in viscosity caused by one mole of CS was 53 times higher than that of PVA.

Thus, in the PVA–CS solution, CS was the polymer with the main effect that drastically changed the rheological properties of the solution, exerting a direct influence on the fabrication of nanofibers as well as on their morphology and diameter distribution. The increase in the viscosity and electrical conductivity of the solution resulted in fibers that were more nano-sized, but the electrospinning performance was reduced. Therefore, for the fabrication of nanofibers with a higher ratio of CS to PVA, the electrospinning parameters need to be more strictly controlled.

### 3.5. FTIR Spectroscopy

**Figure 9:**
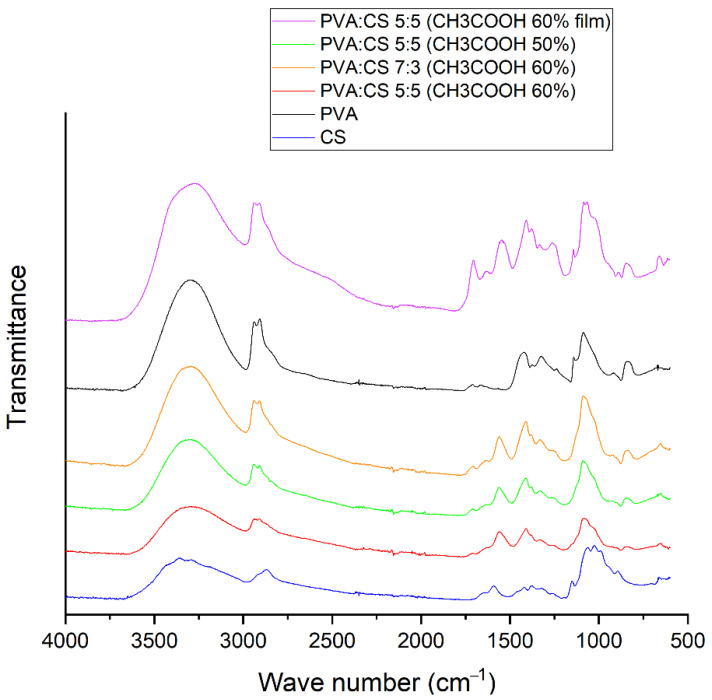
FTIR spectrum of PVA–CS.

To investigate the characteristics of bonds formed in the PVA–CS fiber matrix, infrared spectroscopy of PVA–CS fibers, PVA–CS film (obtained by drying PVA–CS solution), pure PVA powder and pure CS powder was performed (Figure 9).

From Figure 9, it can be easily seen that the IR spectrum of the PVA-CS film contained the peaks with highest intensity. The broad peak with strong intensity in the region of 3000–3600 cm^−1^ showed the abundance of stretching motion of OH and NH bonds present in the PVA–CS film. The simplest spectra were those of the PVA–CS nanofibers, in which the strong peak at position 1705 cm^−1^ corresponded to the stretching of the C=O bond [42] and the peak at 1264 cm^−1^ corresponded to the C–O [43] bond in the carboxyl group of acetic acid; the strong peaks at 1546 cm^−1^ and 1408 cm^−1^ corresponding to the fluctuations of the carboxylate group of the salt [42] completely disappeared.

In general, the spectrum of PVA–CS nanofibers was quite similar to that of pure PVA, but the peaks shifted and changed in intensity depending on the concentrations of the polymers in the system. The higher PVA component in the fiber led to stronger peaks. The two peaks associated with the stretching vibrations of the NH bond and OH bond in the chitosan molecule at position 3362 cm^−1^ and position 3294 cm^−1^ were associated with the wide peak at position 3299 cm^−1^ of the stretching vibration of the OH bond in the PVA molecule into a wide and high intensity peak in the region of 3000–3600 cm^−1^ common to all OH and NH groups in the polymer system. This is similar to observations reported in previous publications [42,43,44,45].

The peaks at 2940 cm^−1^ and 2910 cm^−1^ were related to the asymmetric and symmetric vibrations of CH_2_ stretching, respectively [43,45]. The vibration of the C–H alkyl bond in the chitosan molecule at the position 2869 cm^−1^ was mixed and transformed into a shoulder in the spectrum of the polymer blend; this transition has been confirmed by a previous publication [42].

Peaks 1709 cm^−^^1^ and 1659 cm^−^^1^ were related to the stretching vibrations of the C=O and C–O bonds present in the remaining acetate units in the PVA molecule [45,46] which were still visible in the spectrum of PVA–CS fibers. The peak at 1425 cm^−^^1^ referred to the vibrations of the C–H bond of the methyl group (–CH_3_). The peak at 1089 cm^−^^1^ was caused by the asymmetric stretching vibration of the C–O bond of the acetate group. The peak at 844 cm^−^^1^ was associated with the bending vibrations of C–H bonds in the molecule. These peaks are in agreement with previous statements about PVA [20,25,47,48].

A band at 1589 cm^−^^1^ in the spectrum of chitosan corresponded to the N–H bond bending of the basic amine. The C–H bending and C–N symmetry deformations of chitosan were confirmed by the presence of bands around 1423 cm^−^^1^, 1375 cm^−^^1^ and 1322 cm^−^^1^, respectively. The absorption band at 1153 cm^−^^1^ may have been due to asymmetric stretching of the C–O–C bridge in the glucose group. The bands at 1066 cm^−^^1^ and 1028 cm^−^^1^ corresponded to the stretching of the C–O bonds. Ring stretching corresponded to the 896 cm^−^^1^ peak of OH out-of-plane and the 690 cm^−^^1^ peak of N–H twist vibrations. These peaks are in accordance with previous statements about PVA [49,50,51].

The peaks in the 1590–890 cm^−^^1^ region of the individual polymers were more complex than those obtained from the PVA–CS nanofibers. In general, the peak position of PVA had a slight shift, the wavenumber decreased, and the peaks of chitosan became shoulder shaped. The OH out-of-plane vibrations and the torsional vibrations of the NH group of chitosan also disappeared. This showed that the mobility of the OH and NH_2_ groups of chitosan were no longer available. These changes together with the broadening of the absorption band in the region of 3000–3600 cm^−^^1^ indicated that many hydrogen bonds had formed between PVA and CS molecules. Previous publications also reached similar conclusions [44,45].

## 4. Conclusions

The investigation of nanofiber fabrication and rheological properties of the solutions containing PVA and CS polymers together with acetic acid as solvent showed that these factors were closely related to each other. Any increase in the concentration of either the acetic acid or the polymers led to an increase in the solution’s viscosity. While PVA and acetic acid reduced the conductivity of the solution, CS greatly increased the solution’s conductivity. For this reason, it is easier to obtain fibers from solutions with a high concentration of PVA; however, the fibers obtained from these solutions have larger sizes and more defects. In contrast, although requiring more precise electrospinning parameters, solutions with high CS content resulted in more nanoscale fibers. 

Infrared spectroscopy confirmed the complete separation of the acetic acid from the nanofibers, ensuring its safety for future drug integration purposes. The results of infrared spectrum analysis also showed that hydrogen bonds were the only type of bond formed between the polymers in the nanofibers. 

PVA–CS nanofibers were successfully fabricated from a solution containing 5% (*w*/*w*) PVA; 5% (*w*/*w*) CS; and 60% (*w*/*w*) CH_3_COOH. The following electrospinning parameters were used: voltage 30 kV, feed rate 0.2 mL/h, needle-collector distance 140 mm. The obtained fibers exhibited diameters that were in the range of 77–292 nm, with an average diameter of 153 nm. These nanofiber systems had relatively few defects and could be used for the integration of drugs.

The formed PVA–CS nanofiber system holds promise as a potential material for the integration of therapeutic molecules.

## Figures and Tables

**Table 1:** Results of fiber formation in the electrospinning experiments from PVA, CS 2% (*w*/*w*) in CH_3_COOH 30% (*w*/*w*) solutions.

Needle-Collector Distance (mm)	Feed Rate (mL/h)	PVA: CS Ratio											
		7: 2				8: 2				9: 2			
Voltage (kV)		18	20	25	30	18	20	25	30	18	20	25	30
**150**	0.1	o	+	+	+	o	+	+	+	o	o	+	+
	0.2	o	+	+	+	o	+	+	+	o	o	+	+
	0.3	o	o	*	+	o	+	+	+	o	o	+	+
	0.4	o	o	o	+	o	+	+	+	o	o	*	+
**140**	0.1	o	+	+	+	o	+	+	+	o	+	+	+
	0.2	o	o	+	+	o	o	+	+	o	o	+	+
	0.3	o	o	+	+	o	o	+	+	o	o	+	+
	0.4	o	o	o	+	o	o	+	+	o	o	*	+

(+) Nanofiber formation without drops. (o) Nanofiber and drop formation. (*) Nanofiber formation, but the electrospinning process was unstable.

**Table 2:** Results of the investigation of fiber formation from the electrospinning experiment using PVA 8% (*w*/*w*) and CS 2% (*w*/*w*) with different concentrations of CH_3_COOH.

Needle-Collector Distance (mm)	Feed Rate (mL/h)	C _CH3COOH_ (%, *w*/*w*)													
		30			40			50			60			70	90
Voltage (kV)		18	20	25	18	20	25	18	20	25	18	20	25	(1)	(2)
**150**	0.1	o	+	+	o	+	+	*	+	+	*	+	+	-	-
	0.2	o	+	+	o	+	+	+	+	+	+	+	+	-	-
	0.3	o	+	+	o	o	+	+	+	+	+	+	+	-	-
	0.4	o	+	+	o	o	+	+	+	+	+	*	+	-	-
**140**	0.1	o	+	+	o	+	+	+	+	+	+	+	+	-	-
	0.2	o	o	+	o	o	+	+	+	+	+	+	+	-	-
	0.3	o	o	+	o	o	+	+	*	+	+	+	+	-	-
	0.4	o	o	+	o	o	+	+	+	+	+	+	+	-	-

(1) The solution is too viscous to conduct the electrospinning. (2) No solution is obtained because PVA polymer particles are insoluble in pure acetic acid while CS forms salts with it. (+) Nanofiber formation without drops. (o) Nanofiber and drop formation. (*) Nanofiber formation, but the electrospinning process was unstable.

**Table 3:** Results of investigation of nanofiber fabrication from polymer solutions with different ratios of PVA:CS in 50% (*w*/*w*) and 60% (*w*/*w*) CH_3_COOH solutions.

Feed Rate (mL/h)	PVA:CS Ratio	C_CH3COOH_ (%, *w*/*w*)													
		50%							60%						
Voltage (kV)		18	20	25	27	28	29	30	18	20	25	27	28	29	30
**0.1**	**8:2**	+	+	+	+	+	+	+	+	+	+	+	+	+	+
	**7:3**	+	+	+	+	+	+	+	+	+	+	+	+	+	+
	**6:4**	+	+	+	+	+	+	+	+	+	+	+	+	+	+
	**5:5**	O	o	o	o	o	o	*	*	*	*	*	*	*	*
**0.2**	**8:2**	+	+	+	+	+	+	+	+	+	+	+	+	+	+
	**7:3**	o	+	+	+	+	+	+	+	+	+	+	+	+	+
	**6:4**	o	+	+	+	+	+	+	o	o	*	+	+	+	+
	**5:5**	O	o	o	o	o	o	*	*	*	*	*	*	*	*
**0.3**	**8:2**	+	+	+	+	+	+	+	+	+	+	+	+	+	+
	**7:3**	o	+	+	+	+	+	+	+	+	+	+	+	+	+
	**6:4**	o	*	+	+	+	+	+	o	o	*	+	+	+	+
	**5:5**	O	O	o	o	o	o	o	*	*	*	*	*	*	*
**0.4**	**8:2**	+	+	+	+	+	+	+	+	+	+	+	+	+	+
	**7:3**	o	+	+	+	+	+	+	+	+	+	+	+	+	+
	**6:4**	o	o	+	+	+	+	+	o	o	*	+	+	+	+
	**5:5**	O	O	o	o	o	o	o	*	*	*	*	*	*	*
**0.1–0.4**	**4:6**	–	–	–	–	–	–	–	–	–	–	–	–	–	–

(+) Nanofiber formation without drops. (o) Nanofiber and drop formation. (*) Nanofiber formation, but the spray of the solution is not stable. (O) Drop formation. (–) The solution is too viscous to conduct electrospinning.

**Table 4:** Morphology and diameter distribution of PVA–CS fibers.

C_CH3COOH_ (% *w*/*w*)	PVA:CS ratio	Mean (nm)	Median (nm)	Min (nm)	Max (nm)	Morphology
**50**	**8:2**	239	232	71	574	Presence of a rather large polymer streak
	**7:3**	213	206	38	418	Presence of small branches, small polymer streaks and curls
	**6:4**	222	215	86	413	Presence of small drops, polymer streaks and curls
	**5:5**	192	192	86	320	Presence of individual small spots
**60**	**8:2**	218	215	109	371	Presence of polymer streaks
	**7:3**	197	192	77	343	Presence of small branches and polymer streaks
	**6:4**	183	185	54	308	Presence of small drops, polymer streaks and curls
	**5:5**	153	153	77	292	Presence of individual small spots

**Table 5:** Conductivity of PVA–CS solutions in 30% (*w*/*w*) acetic acid.

PVA:CS Ratio	7:2	8:2	9:2	10:2
Electrical conductivity (Ɜ) μS/cm	1957	1893	1777	1687

**Table 6:** Conductivity of PVA–CS solutions in 60% (*w*/*w*) acetic acid.

PVA:CS Ratio	9:2	7:3	6:4	5:5
Electrical conductivity (Ɜ) μS/cm	802	1348	1696	2294

**Table 7:** Viscosity of PVA–CS solutions in 30% (*w*/*w*) acetic acid.

PVA:CS Ratio	7:2	8:2	9:2	10:2
Viscosity (ⴄ) (mPa·s)	2224.1	3153.8	4275.6	5470.1

**Table 8:** Viscosity of PVA–CS solutions in 60% (*w*/*w*) acetic acid.

PVA:CS Ratio	9:2	7:3	6:4	5:5
Viscosity (ⴄ) (mPa·s)	7016.4	8575.7	10,148	13,903

## Data Availability

The data presented in this study are available on request from the corresponding author.
